# Supplementary material for: Increased respiratory morbidity associated with exposure to a mature volcanic plume from a large Icelandic fissure eruption
Source: Nat Commun. 2021 Apr 12;12:2161. doi: 10.1038/s41467-021-22432-5 (PMC8042009; doi:10.1038/s41467-021-22432-5)
Supplement: Supplementary file 1 — Supplementary Information [file 41467_2021_22432_MOESM1_ESM.pdf]

Increased respiratory morbidity associated with exposure to a mature volcanic plume from a large Icelandic fissure eruption

Supplementary Information

Hanne Krage Carlsen, Evgenia Ilyinskaya, Peter J. Baxter, Anja Schmidt, Throstur Thorsteinsson, Melissa Anne Pfeffer, Sara Barsotti, Francesca Dominici, Ragnhildur Gudrun Finnbjornsdottir, Thorsteinn Jóhannsson, Thor Aspelund, Thorarinn Gislason, Unnur Valdimarsdóttir, Haraldur Briem, Thorolfur Gudnason

This file contains

Supplementary Figures 1-3

Supplementary Methods

Supplementary Figures

Supplementary Figure 1 Associations between exposure to mature plume and PCMD visits for non-respiratory diagnosis categories (ICD codes and *n* of individuals in brackets). (a-d) show unadjusted results for mature plume exposure. (e-h) show results adjusted for SO_2, an indicator of the primitive plume. (a, e) Nausea and vomiting (R11, *n* = 2,063 individuals); (b, f) Headaches (R51, *n* = 8,269 individuals); (c, g) Circulatory system disease (I, *n* = 21,811 individuals); (d, h) Eye irritation (H10-H11, *n* = 34,699 individuals). Results are reported as percent change in RR with error bars showing the 95% confidence intervals. Results are shown for different age groups (all age groups combined; < 18 years; 18-64 years; > 64 years) for several lag combinations; *n* of individuals in each age group is shown on the x-axis.

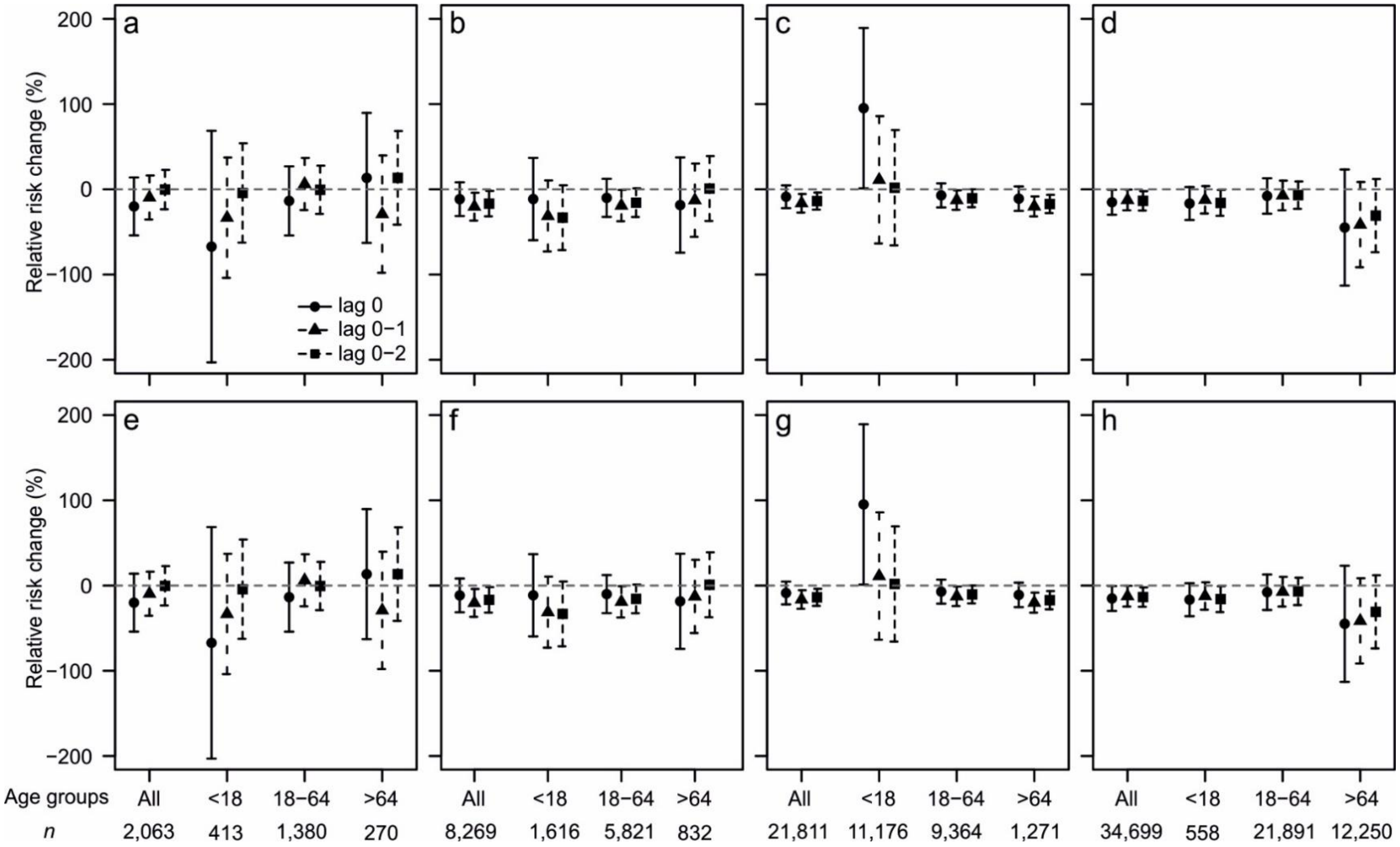

Supplementary Figure 2 Associations between exposure to mature plume and HED visits for non-respiratory diagnosis categories (ICD codes and *n* of individuals in brackets). (a-d) show unadjusted results for mature plume exposure. (e-h) show results adjusted for SO_2, an indicator of the primitive plume. (a, e) Nausea and vomiting (R11, *n* = 1,610 individuals); (b, f) Headaches (R51, *n* = 2,966 individuals); (c, g) Circulatory system disease (I, *n* = 2,045 individuals); (d, h) Eye irritation (H10-H11, *n* = 18,161 individuals). Results are reported as percent change in RR with error bars showing the 95% confidence intervals. Results are shown for different age groups (all age groups combined; < 18 years; 18-64 years; > 64 years) for several lag combinations; *n* of individuals in each age group is shown on the x-axis.

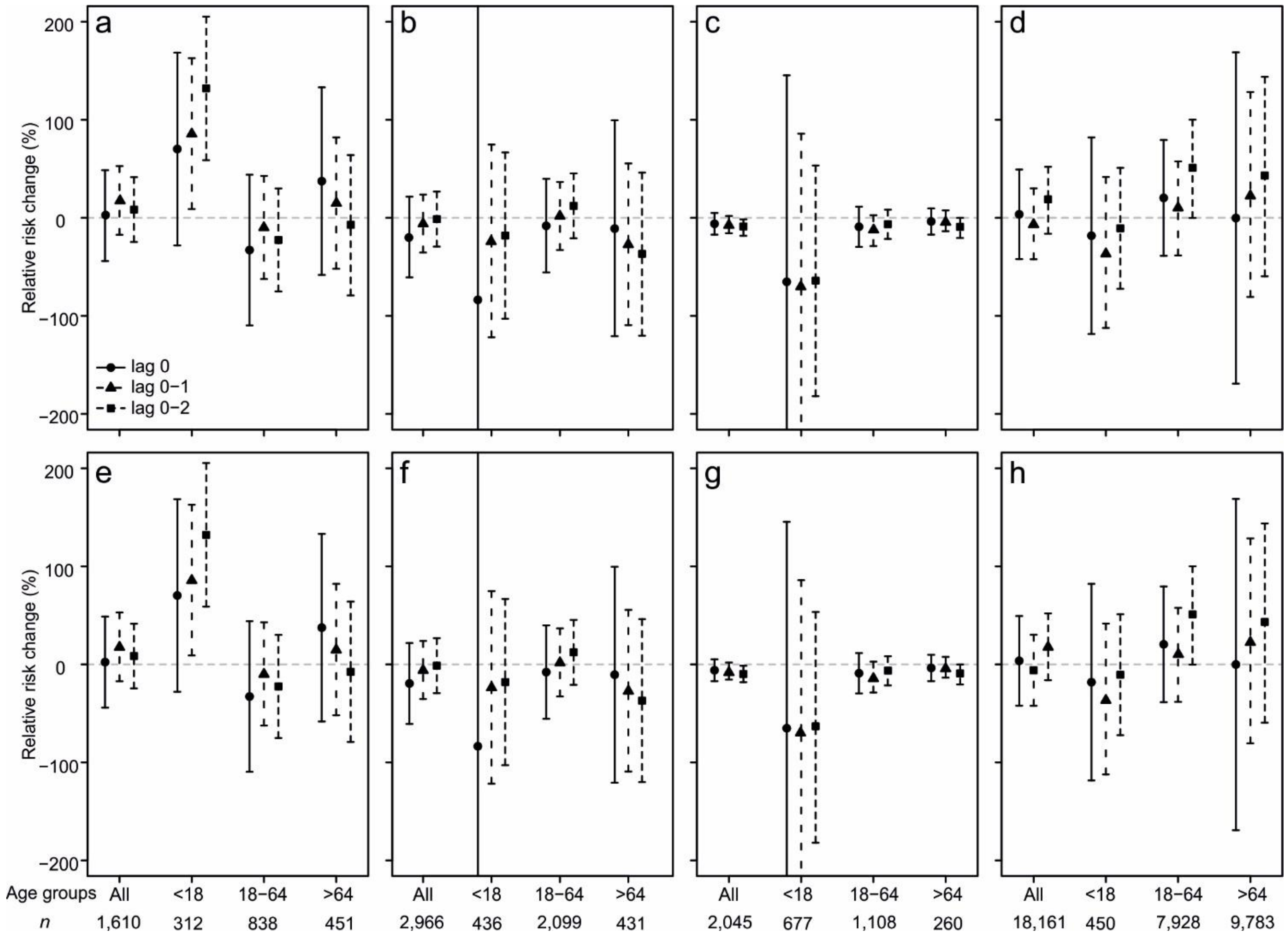

Supplementary Figure 3 Lag-associations for exposure to mature plume and health outcomes the following day, modelled with splines. (a) Association with AMD, *n* = 48,014 individuals; (b) Association with PCMD visits for respiratory diagnoses, *n* = 110,809 individuals; (c) Association with HED for respiratory diagnoses, *n* = 20,725 individuals. Results are reported as change in RR with error bars showing the 95% confidence intervals.

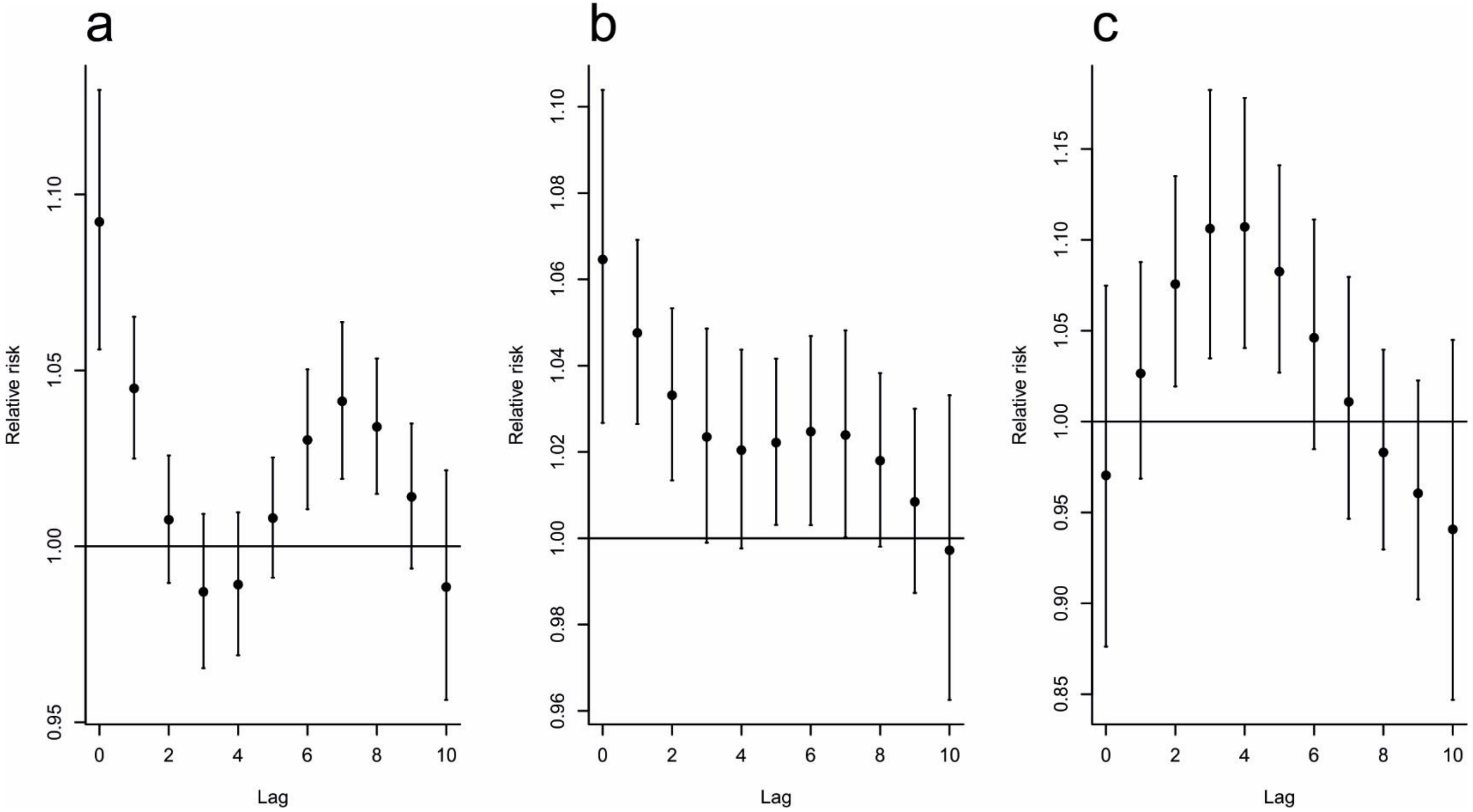

Supplementary Methods

R code model examples

1. Unadjusted results (Figure 2a-c, Figure 4, Supplementary Figure 1a-d, Supplementary Figure 2a-d).

model <- gam(outcome ~ Mature_plume_indicator + as.factor(day_of_week) + odd_holidays + s(day of time series, k=10) + s(day of year, bs="cc") , family=poisson(), data=data)

Where *outcome* is the number of visits or dispensings for the different health outcomes, outcome_lag1 is the outcome at lag 1 (to diminish autocorrelation).

2. SO_2-adjusted results (Figure 2d-f, Supplementary Figure 1e-h, Supplementary Figure 2e-h)

model <- gam(outcome ~ Mature_plume_indicator + SO_2 + as.factor(day_of_week) + odd_holidays + s(day of time series, k=10) + s(day of year, bs="cc") , family=poisson(), data=data)

Where the SO_2 term has the same lag as the mature plume exposure.

For anti-asthma medication dispensing, we use the quasipoisson distribution (*family=quasipoisson ()*)and for PCMD and HED, we use the poisson distribution (*family=poisson()*).

3. Lag-association model comparing mature plume and SO_2 (Figure 3 and Supplementary Figure 3)

#first, a cross basis was created for the mature plume, with lags up to 5 or 10 days, with a linear structure across the variable values and polynomial structure with 4 degrees of freedom for the lag structure.

```
cb.plume<- crossbasis(data_ph$mature plume,lag=5
           , argvar=list(fun="lin")
           , arglag=(list(fun="poly", degree=4)))
```

#Then, a cross basis was created for SO_2, with lags up to 5 or 10 days, with a threshold structure for variable values and polynomial structure with 4 degrees of freedom for the lag structure.

```
cb.so2<- crossbasis(data_ph$so2,lag=10
           , argvar=list("thr",thr.value=c(5))
          , arglag=(list(fun="poly", degree=4)))
```

#A model is constructed

```
 model <- gam(outcome ~cb.plume + cb.so2
          +as.factor(day_of_week)  +s(nday, k=4)
           +oddho+ s(doy3, bs="cc"), data=data, family=quasipoisson())
```

And the outcome of this model is used to predict based

a) for the mature plume crossbasis

```
pred.hgJ12<- crosspred(cb.plume,model, at=0:1)
```

b) the SO_2 crossbasis to the value 125.

```
pred.hgJ12_so2 <- crosspred(cb.so2,model,at=0:125)
```

And then extracted and plotted using "plot".
