# Supplementary material for: Increased respiratory morbidity associated with exposure to a mature volcanic plume from a large Icelandic fissure eruption
Source: Nat Commun. 2021 Apr 12;12:2161. doi: 10.1038/s41467-021-22432-5 (PMC8042009; doi:10.1038/s41467-021-22432-5)
Supplement: Supplementary file 3 — Description of Additional Supplementary Files [file 41467_2021_22432_MOESM3_ESM.pdf]

Description of Additional Supplementary Files

File Name: Supplementary Movie 1
Description: Model-simulated hourly-mean sulphur dioxide (SO_2) and sulphate aerosol (SO_4) ground-level mass concentrations (µg/m^3) for the eruptive period 31 August 2014 to 30 September 2014 based on model simulations described in Schmidt et al., 2015. The animation has been previously published in Ilyinskaya et al., 2017
