# Supplementary figures and images for: Increased respiratory morbidity associated with exposure to a mature volcanic plume from a large Icelandic fissure eruption
Source: Nat Commun. 2021 Apr 12;12:2161. doi: 10.1038/s41467-021-22432-5 (PMC8042009; doi:10.1038/s41467-021-22432-5)

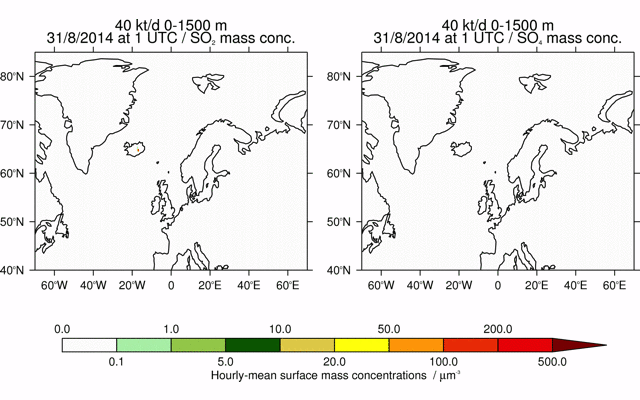

Supplement: Supplementary file 4 — Supplementary Movie 1 [file 41467_2021_22432_MOESM4_ESM.gif]
