# Supplementary material for: Increased respiratory morbidity associated with exposure to a mature volcanic plume from a large Icelandic fissure eruption
Source: Nat Commun. 2021 Apr 12;12:2161. doi: 10.1038/s41467-021-22432-5 (PMC8042009; doi:10.1038/s41467-021-22432-5)
Supplement: Supplementary file 5 — Reporting Summary [file 41467_2021_22432_MOESM5_ESM.pdf]

nature research

Corresponding author(s): Hanne Krage Carlsen, Evgenia Ilyinskaya

Last updated by author(s): 17 February 2021

Reporting Summary

Nature Research wishes to improve the reproducibility of the work that we publish. This form provides structure for consistency and transparency in reporting. For further information on Nature Research policies, see our Editorial Policies and the Editorial Policy Checklist.

Statistics

For all statistical analyses, confirm that the following items are present in the figure legend, table legend, main text, or Methods section.

n/a	Confirmed	
☐	☒	The exact sample size (*n*) for each experimental group/condition, given as a discrete number and unit of measurement
☐	☒	A statement on whether measurements were taken from distinct samples or whether the same sample was measured repeatedly
☐	☒	The statistical test(s) used AND whether they are one- or two-sided<br>*Only common tests should be described solely by name; describe more complex techniques in the Methods section.*
☐	☒	A description of all covariates tested
☒	☐	A description of any assumptions or corrections, such as tests of normality and adjustment for multiple comparisons
☐	☒	A full description of the statistical parameters including central tendency (e.g. means) or other basic estimates (e.g. regression coefficient) AND variation (e.g. standard deviation) or associated estimates of uncertainty (e.g. confidence intervals)
☐	☒	For null hypothesis testing, the test statistic (e.g. *F*, *t*, *r*) with confidence intervals, effect sizes, degrees of freedom and *P* value noted<br>*Give P values as exact values whenever suitable.*
☒	☐	For Bayesian analysis, information on the choice of priors and Markov chain Monte Carlo settings
☐	☒	For hierarchical and complex designs, identification of the appropriate level for tests and full reporting of outcomes
☐	☒	Estimates of effect sizes (e.g. Cohen's *d*, Pearson's *r*), indicating how they were calculated

Our web collection on statistics for biologists contains articles on many of the points above.

Software and code

Policy information about availability of computer code

Data collection	Data were not collected by the authors of this study. Data were extracted from preexisting databases as Microsoft Office Excel files.
Data analysis	Air quality data were analysed with Microsoft Office Excel 2010<br>Health records analysed with Rstudio desktop (version 1.2.5), R (version 3.6), library "mgcv" (version 1.8-27).

For manuscripts utilizing custom algorithms or software that are central to the research but not yet described in published literature, software must be made available to editors and reviewers. We strongly encourage code deposition in a community repository (e.g. GitHub). See the Nature Research guidelines for submitting code & software for further information.

Data

Policy information about availability of data

All manuscripts must include a data availability statement. This statement should provide the following information, where applicable:

- Accession codes, unique identifiers, or web links for publicly available datasets
- A list of figures that have associated raw data
- A description of any restrictions on data availability

The health record data that support the findings of this study can be made available to researchers after approval of a formal application to the Icelandic Directorate of Health and the Icelandic Bioethics committee. The data are not publicly available due to them containing sensitive individual-level information.

The SO2, PM2.5 and SO42- data that support the findings of this study are available from the Environment Agency of Iceland but restrictions apply to the availability of these data, which were used under license for the current study, and so are not publicly available. Data are however available from the authors upon reasonable request and with permission of the Environment Agency of Iceland.

The results of the Icelandic Meteorological Office online questionnaire that support the findings of this study are available from the Icelandic Meteorological Office but restrictions apply to the availability of these data, which were used under license for the current study, and so are not publicly available. Data are however available from the authors upon reasonable request and with permission of the Icelandic Meteorological Office.

Field-specific reporting

Please select the one below that is the best fit for your research. If you are not sure, read the appropriate sections before making your selection.

☐ Life sciences ☐ Behavioural & social sciences ☒ Ecological, evolutionary & environmental sciences

For a reference copy of the document with all sections, see nature.com/documents/nr-reporting-summary-flat.pdf

Ecological, evolutionary & environmental sciences study design

All studies must disclose on these points even when the disclosure is negative.

Study description

Time series analysis of air quality.
Two types of data were considered: air quality and population health records from Reykjavik, Iceland

1) Exposure data: The air pollutants considered were gaseous sulphur dioxide (SO2), fine particulate matter (PM2.5) and particulate sulphate (SO42-).

2) Health outcome data. The following respiratory health indicators were considered: daily number of asthma medicine dispensings; number of individuals with respiratory health diagnoses, and other diagnoses thought to be of relevance (nausea, eye irritation, cardiovascular disease, and headaches) in primary care; and number of individuals with respiratory health diagnoses in the hospital emergency room.
The outcomes were stratified by age and diagnostic categories in several types of regression analysis.

Research sample

Air quality data: Air pollutant data in Iceland, collected by the Environment Agency of Iceland (EAI) operational air quality monitoring network. Airborne concentrations of SO2 and PM2.5 are automatically measured and recorded, with the results available in real-time (loftgaedi.is). Samples of airborne sulphate were collected using a filter sampler. The filters were changed once every 24 hours. After sampling, the filters were analysed for sulphate ion concentration using ion chromatography.

Health records: Our case study population is Greater Reykjavík, Iceland's capital region which comprises the capital city Reykjavík and 6 municipalities around it, collectively called Reykjavík here. Reykjavík contains ~60% of Iceland's total population. It had 205,282 residents at the beginning of the study period, and 215,965 residents at the end. The Icelandic health care system is state-centred, mainly publicly funded system with universal coverage. Data were accessed through national centralised records. We obtained data on respiratory health and the other relevant diagnoses (nausea, eye irritation, cardiovascular disease, and headaches) and individual data on residence (postcode), age, sex and an anonymous personal identification number from 1) the National Medicines Register; 2) Primary care centres (that function as first point of contact) and 3) Landspítali, the national university hospital, the country's centre of clinical excellence. All 3 registers are held by the Icelandic Directorate of Health and extraction is subject to approval from the Icelandic Bioethical Committee.

Sampling strategy

Air quality data: The data analysed in this study were collected at two EAI stations, Hvaleyrarholt (HVAL) and Grensásvegur (GREN). The two stations were selected based on following criteria. HVAL is the only EAI station that measured sulphate PM (by filter sampling, 24-hour mean value), in addition to measurements of gaseous pollutants including SO2 (up to 1 min time series resolution) and PM2.5. HVAL was therefore used to calculate the ratio of SO42- to SO2. GREN monitors only the gaseous pollutants and PM2.5, but was selected for this study in addition to HVAL due to its more central location than HVAL, it is considered to be more representative than HVAL of the population exposure to SO2 due to its location (central vs rural, respectively). GREN data were therefore used for calculating the health effects associated with SO2 exposure. There was a good correlation between SO2 time series at HVAL and GREN: Pearson's correlation coefficient = 0.966, p <0.001 during the eruption.

Health records: The study population included all individuals who fulfilled the following 3 criteria. 1) were dispensed asthma medication (AMD), or received a respiratory disease,(ICD code J) or other relevant disease (nausea, eye irritation, cardiovascular disease, and headache) at an MD visit at a primary care clinic (MDPC), or the Landspitali emergency room (HED) in Iceland, 2) used healthcare services between 1 January 2010 and 31 December 2014 and 3) had a legal residency in Reykjavík.

Data collection

Air quality data: The air quality stations are operated, calibrated and maintained by the EAI, the institute mandated to monitor air quality in Iceland. The sulphate sample extraction and analysis were done at the Innovation Centre of Iceland on behalf of EAI.

Health records: All registers are held by the Icelandic Directorate of Health and extraction is subject to approval from the Icelandic Bioethical Committee. We obtained data on respiratory health and the other relevant diagnoses (nausea, eye irritation, cardiovascular disease, and headaches) and individual data on residence (postcode), age, sex and an anonymous personal identification number from 1) the National Medicines Register; 2) Primary care centres (that function as first point of contact) and 3) Landspítali, the national university hospital, the country's centre of clinical excellence.

From the National Medicines Register we extracted data on dispensing (pharmacy sales to individuals) of prescription anti-asthma medication (AMD) classified by The World Health Organisation Anatomical Therapeutic Chemical code R03. From the primary care centres and hospital emergency department (HED) databases at the Directorate of Health we extracted data on individuals diagnosed

	with respiratory diseases and other relevant conditions as listed above
Timing and spatial scale	Spatial scale: Greater Reykjavík, Iceland's capital region which comprises the capital city Reykjavík and 6 municipalities around it. In this study, it is referred to as Reykjavík.<br><br>Timing:<br>Air quality data: Analysed time series of daily mean of SO2 and PM2.5 was 1 January 2010 to 31 December 2014. Analysed time series of SO42- was 2 January 2014 to 31 December 2014. The focus of this study was Holuhraun eruption period 31 August 2014 - 27 February 2015. The time period before 31 August 2014 was used as a reference period for local air quality without an input from a volcanic source. The start of the time series for SO2 and PM2.5 (2 Jan 2012) was selected based on the fact that there were no volcanic eruptions in Iceland during the years 2012 and 2013. The start of the time series for SO42- (2 Jan 2014) was determined by the fact that this is when SO42- sampling began. The end of the time series (31 Dec 2014) was determined by the availability of the co-analysed public health data (see below). This is not considered to significantly impact the conclusions of this study. The eruption intensity waned significantly after the first 4 months and only 5 out of the 38 volcanogenic pollution days occurred in January-February 2015 (references in manuscript)<br><br>Health records: The study period was 1 January 2010 to 31 December 2014, which was selected based on the availability of health records. The Holuhraun eruption took place between 31 August 2014 and 27 February 2015. The time before the eruption was used a reference period. The study period covers only 4 out of the 6 months of the eruption due to a change in the database recording of events. As mentioned under Air quality data, this is not considered to impact the results.
Data exclusions	Air quality data: 29 December to 1 January each year was excluded from all calculations of SO42- due to noticeable New Year's Eve firework/bonfire pollution<br><br>Health records: In the health data, individuals with no registered postcode, or a postcode outside the capital area were excluded.<br><br>The exclusion criteria were pre-established
Reproducibility	n/a. The study was an observational study, treating the volcanic eruption as a natural experiment exposing a population to volcanogenic air pollutants
Randomization	n/a. This was an observational study and therefore no randomisation was possible.
Blinding	n/a. Given the observational nature of the study, blinding the study subjects or researchers completely to their exposure status was not possible. We address the issue of subjects' awareness of exposure status by analysing separately the health care usage during the episodes where the public was "blinded" to exposure due to a lack of public advisories about presence of volcanogenic pollution

Did the study involve field work? ☐ Yes ☒ No

Reporting for specific materials, systems and methods

We require information from authors about some types of materials, experimental systems and methods used in many studies. Here, indicate whether each material, system or method listed is relevant to your study. If you are not sure if a list item applies to your research, read the appropriate section before selecting a response.

Materials & experimental systems

n/a	Involved in the study	
☒	☐	Antibodies
☒	☐	Eukaryotic cell lines
☒	☐	Palaeontology and archaeology
☒	☐	Animals and other organisms
☐	☒	Human research participants
☒	☐	Clinical data
☒	☐	Dual use research of concern

Methods

n/a	Involved in the study	
☒	☐	ChIP-seq
☒	☐	Flow cytometry
☒	☐	MRI-based neuroimaging

Human research participants

Policy information about studies involving human research participants

Population characteristics	The total study population was 8,014 individuals, or a mean value of 131 (SD 70) per day for asthma medication dispensing, 119,948 individuals, a mean value of 142 (SD 91) per day, for PCMD with a respiratory diagnosis, and 20,725 individuals, or a mean value of 18 (SD 8) per day, for HED with respiratory diagnosis.<br>The results are reported by age groups (<18 years, 18-64 years, and >64 years) whose sizes are presented with the results (Tables 2,3,4).<br>AMD: <18 years, n= 15,064; 18-64 years, n= 25,461; >64 years, n=7,489,<br>Respiratory PCMD: <18 years, n=34,051; 18-64 years, n= 72,629; >64 years, n= 12,368I,<br>Respiratory HED: <18 years, n=7,260; 18-64 years, n= 8,767; >64 years, n=4,698.<br>Results by sex are not reported.<br>Other non-respiratory diagnoses were reported in the supplement with the following descriptive statistics daily mean (±SD) of HED for R11 (Nausea and vomiting): 1.1 (±1.1), R51 (Headaches): 1.9 (±1.5), I (Circulatory system disease: 21.2 (10.9), and H10-H11 (Eye irritation): 1.2 (±1.1).
Recruitment	n/a. Data accessed through national centralised records
Ethics oversight	The Icelandic Bioethics committee (ref. no. for the current study: VSNb2015050022/03.01)

Note that full information on the approval of the study protocol must also be provided in the manuscript.
